# Partnering With Title VI Directors to Put Survey Data to Use in Improving the Health and Well-Being of Native Elders

**DOI:** 10.1093/geroni/igaa057.322

**Published:** 2020-12-16

**Authors:** Collette Adamsen, Ramona Danielson

**Affiliations:** 1 University of North Dakota, Grand Forks, North Dakota, United States; 2 North Dakota State University, Fargo, North Dakota, United States

## Abstract

Through Title VI of the Older Americans Act, federally recognized American Indian tribes, Alaska Native villages, and Native Hawaiians are eligible for grant funding to promote elders’ access to nutritious food and address other community-specific needs, such as transportation. Title VI directors are often short-staffed and work flexibly and creatively to accomplish administrative tasks as well as direct care for elders. The National Resource Center on Native American Aging (NRCNAA) developed the “Identify our Needs: A Survey of Elders”, conducted with adults ages 55+ every three years since 1999, to help directors fulfill grant-required requirements to report on community needs assessment data about elders’ needed services and health status. Directors administer the survey; responses are then scanned and analyzed by NRCNAA, with a site-specific summary returned to directors, at no cost to the site. To learn more about how survey data are utilized, NRCNAA staff interviewed four Title VI directors from across the US in 2018. In addition to meeting the needs assessment requirement, directors use the data to seek resources and support, build collaborative relationships, and evaluate and build their programs. Conducting the survey has allowed for discussions with elders about dimensions of elder health, advanced medical directives, and medical power of attorney. Additionally, specific interventions have been designed to address needs identified by the survey, including diabetic foot care, yoga, and falls prevention. Through the survey, the NRCNAA is able to support many dedicated Title VI directors working to improve the health and well-being of Native elders.

